# Bilateral renal ischemia after kyphoplasty and clodronate treatment: a case report

**DOI:** 10.1186/1752-1947-8-76

**Published:** 2014-02-26

**Authors:** Angela Notarnicola, Giuseppe Maccagnano, Alessio Casalino, Lorenzo Moretti, Andrea Piazzolla, Biagio Moretti

**Affiliations:** 1 Department of Medical Science of Base, Neuroscience and Organs of Sense, Orthopedics Section, Faculty of Medicine and Surgery of University of Bari, General Hospital, Piazza Giulio Cesare 11, 70124 Bari, Italy; 2 Department of Medical Science of Base, Neuroscience and Organs of Sense, Course of Motor and Sports Sciences, Faculty of Medicine and Surgery, University of Study of Bari, Lungomare Starita 1, 70123 Bari, Italy

**Keywords:** Drug-induced vasculitis, Clodronate, Bilateral renal ischemia, Lumbar vertebral fracture, Kyphoplasty

## Abstract

**Introduction:**

The most common adverse effects associated with bisphosphonates are renal toxicity, acute-phase reactions, gastrointestinal toxicity, osteonecrosis of the jaw, transitory fever and uveitis. We report a unique adverse case of vasculitis induced by clodronate.

**Case presentation:**

A 61-year-old Caucasian woman developed bilateral renal ischemia after kyphoplasty and clodronate treatment for lumbar vertebral fracture. Tests revealed a vasculitis due to clodronate treatment. The antithrombotic and immunosuppressive drugs allowed us to reduce the extent of the renal ischemia. In the following months the increased auto-antibodies returned to the healthy physiological range, but the chronic renal failure persisted.

**Conclusions:**

Drug-induced vasculitis is an inflammation of blood vessels caused by the use of various pharmaceutical agents. The spectrum of drug-induced vasculitis can range from cutaneous rashes to fatal multi-organ involvement. To the best of our knowledge this is the first documented case of drug-induced vasculitis caused by clodronate in the literature. Previously, it was verified that clodronate injection could increase the pro-apoptotic action on immune cells. Further studies are necessary to clarify the role of bisphosphonates on drug-inducing vasculitis.

## Introduction

Clodronic acid, a first-generation bisphosphonate, has been successfully used in the treatment of high bone-turnover states, Paget’s disease and osteolytic bone metastases [1]. It is a synthetic analogue of pyrophosphate, an endogenous regulator of bone mineralization, which contains two phosphanate groups that allow recognition by osteoclasts and macrophage membranes [2]. The interaction between osteoclasts and clodronic acid is responsible for the inhibition of the ADP and/or ATP (adenosine-diphosphate and/or adenosine-5′-triphosphate) mitochondrial translocation activity, bone remodeling of the matrix and may also induce apoptosis [3]. The clodronic acid is able to develop an anti-inflammatory action through the inhibition of I-κB enzyme which is in the macrophage cytoplasm and is responsible for the release of interleukin-1 beta, interleukin-6 and tumor necrosis factor-alpha, all of which play a central role in bone remodeling. Furthermore, clodronic acid induces an activation of an inflammatory cascade and attracts new leukocytes to the inflammation [4,5]. The most common adverse effects associated with bisphosphonates are renal toxicity, acute-phase reactions, gastrointestinal toxicity and osteonecrosis of the jaw. Less frequently there are cases of transitory fever and uveitis [6-8].

Our case documents a developing progressive necrosis of both kidneys after clodronic injection until now never described in the literature. Our clinical research allowed us to make a new pathogenetic hypothesis.

## Case presentation

A 61 year-old Caucasian woman (height 150cm, weight 50Kg), in good general health, suffered a lumbar spine trauma caused by a fall in her home. The clinical picture, X-ray and CT (computed tomography) scan showed a fracture of the L2 vertebral body. We performed a kyphoplasty with high density polymethylmethacrylate cement (PMMA) (X-Pid, Medtronic Inc., Minneapolis, Minnesota, USA). The dual-energy X-ray absorptiometry (DEXA) exam confirmed the osteoporotic picture (T-score −4 DS). At that point we started an osteoporosis treatment. Considering the DEXA results and clinical signs of the patient, a bisphosphonate was administered [9]. To treat the post-traumatic low back pain we started with clodronic acid (200mg once every two weeks) through intramuscular injection (Clasteon®, Abiogen Pharma S.p.A., Pisa, Italy) with the intention of substituting the clodronic acid with one of the other bisphosphonates (alendronic acid, risedronic acid, ibandronic acid) by oral intake. In fact, clodronic acid inhibits bone resorption and possesses good anti-inflammatory and analgesic properties, useful in the acute post-traumatic phase [10,11].

**Figure 1:**
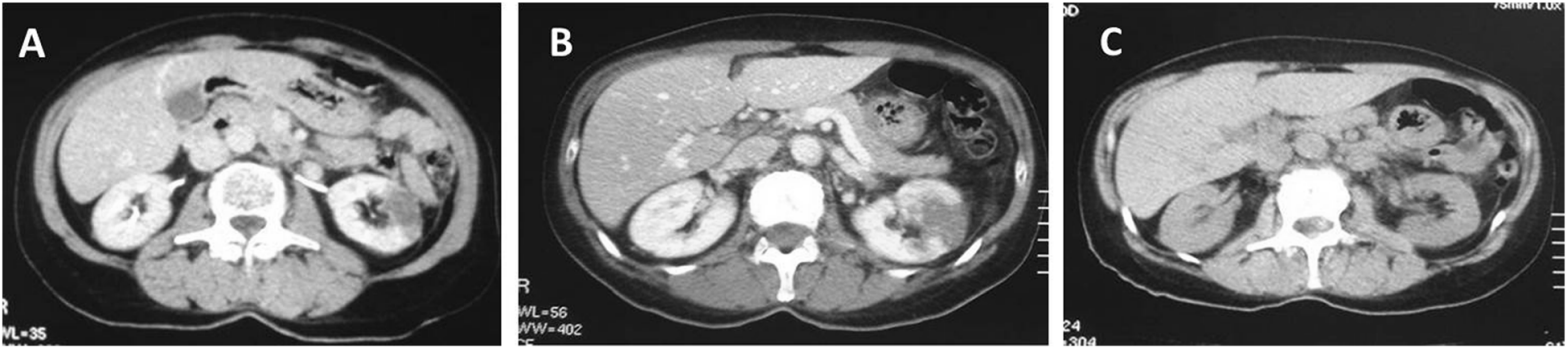
**Computed tomography imaging.** The first computed tomography. An ischemic area in the left kidney **(A)**. The computed tomography a few days later. An increasing of left renal ischemia tissue and a novel ischemia area in the right kidney **(B)**. The follow-up computed tomography after one year. A reduction of ischemic areas in both kidneys **(C)**.

In the weeks following discharge, our patient began to complain of fever, arthritis, low back pain and acute abdominal pain and was hospitalized. A thorax-abdomen-pelvic CT scan showed a left kidney ischemia (Figure 1A). At first, we suspected that the renal artery might have been closed by the PMMA cement used in her surgery and that this may have been responsible for an artery thrombosis. Contextually, we suspended the clodronate treatment and we started anti-thrombotic therapy with enoxaparin sodium (Clexane®, Sanofi-Aventis S.p.A., Milan, Italy) (4000IU once for two times a day for 30 days) and ticlopidine (Ticlid®, Sanofi-Aventis S.p.A, Milan, Italy) (200mg tablets two times a day) after the suspension of enoxaparin. We admitted the patient to hospital to complete the diagnostic procedures (Tables 1 and 2). In the following days the blood tests showed positivity for anti-neutrophil cytoplasmic antibody (ANCA), anti-double-stranded deoxyribonucleic acid (DNA) antibody (anti-ds DNA), anti-nuclear auto-antibodies (ANA) and anti-myeloperoxidase antibodies (MPO). At the same time, the CT scan showed an increase in left renal ischemic tissue and a new ischemia area in the right kidney (Figure 1B). An arteriography or a kidney biopsy would have been useful to make a diagnosis. Considering kidney blood tests it was not possible to carry out the arteriography due to the risk of inducing a kidney failure [12]. The patient did not give the consent to perform a kidney biopsy.

**Table 1:** The values of blood and urine tests

	**Values at admission**	**Values during the hospital stay**	**Physiological range**
**Blood tests**			
Erythrocytes	3.41×10^6^/ul	3.35×10^6^/ul	3.75 to 4.54×10^6^/ul
Leukocytes	7.03×10^3^/uL	5.26×10^3^/uL	4.0 to 9.32×10^3^/uL
Platelets	336×10^3^/uL	345×10^3^/uL	150 to 500×10^3^/uL
Creatinine	1.03mg/dL	1.00mg/dL	0.51 to 0.95mg/dL
GFR (glomerular filtration rate)	57mL/minute	61mL/minute	>90mL/minute
Urea	34mg/dL	38mg/dL	15 to 38mg/dL
Total bilirubin	0.30mg/dL	0.30mg/dL	0.20 to 1.00mg/dL
S-Aspartate Aminotransferase (AST)	12U/L	17U/L	15 to 37U/L
S-Alanine Aminotransferase (ALT)	20U/L	22U/L	12 to 78U/L
S-Gamma Glutamine Transpepetidase (GGT)	25U/L	25U/L	5 to 55U/L
Albumin	3.4g/dL	3.3g/dL	3.4 to 5.0g/dL
Prothrombin time (PT)	1.07 ratio	1.04 ratio	<1.20
Fibrinogen	365mg/dL	431mg/dL	200 to 400mg/dL
Activated partial thromboplastin time (aPTT)	1.21 ratio	1.17 ratio	<1.20
Thrombin Time (TT)	1.12 ratio	1.07 ratio	<1.20
D-dimers (DD)	0.7mg/dL	1.17mg/dL	<0.5mg/dL
Lactate dehydrogenase (LDH)	-	348U/L	84 to 246U/L
S-anti-dsDNA autoantibodies	-	63.5U/mL	<25U/mL (chemiluminescent assay)
S-anti-nuclear autoantibodies (ANA)	-	Positivity, pattern nuclear speckled title 1/80	(immunofluorescent assay)
S-Autoantibodies anti-myeloperoxidase (MPO)	-	58mm/h	1 to 20mm/h (immunoenzymatic assay)
Anti-neutrophil cytoplasmic antibodies (ANCA)	-	Positive	Negative (immunofluorescent assay)
S-Autoantibodies to extractable nuclear antigens (ENA)	-	<0.5	Negative <1 (chemiluminescent assay)
S-Autoantibodies anti-smooth muscle (ASMA)	-	Negative	Negative (immunofluorescent assay)
**Urine exam**			
Hemoglobin (Hb)	-	>0.6mg/dl	0 to 0.02
Proteins	-	0	0
Sediment	-	5 to 10 red blood cells for field	-

**Table 2:** Image techniques performed during the hospital stay

**Instrumental exam**	**Results**
Thorax X-rays	No pathological tissue signs and pleural effusion. Normal volume of heart.
echocardiography	Left ventricle is normal (ejection fraction = 60%). It shows a low left atrial dilatation with aortic and mitral insufficiency. There is no pericardial effusion.
Abdomen ultrasound	The abdominal organs are normally. There is no artery disease; in fact, the hepatic, kidney and spleen flow and peripheral resistance are physiologic.
Thorax abdomen pelvic computed tomography (CT) scans	The organs are normal.
	Left kidney: reduction of the extension of previous necrosis area.
	Right kidney: new ischemic area at the inferior tip.

Although we suspected a link between the extension of renal ischemia on the right kidney to the first thrombotic event, the antibodies’ positivity supported a diagnosis of immunological vasculitis, specifically systemic lupus erythematosus (SLE). We, therefore, started an immunosuppression therapy with cyclophosphamide (Endoxan®, Baxter S.p.A., Rome, Italy) (50mg, once a day, four days a week). The follow-up blood test to check anti-inflammatory and antibody parameters showed a progressive return to normality, which was achieved by the six month check-up.

However, the good clinical response to immunosuppressive therapy, the absence of clinical signs of SLE and physiological range of antibodies allowed us to exclude the previous immunological hypotheses. We suspected, therefore, that the vasculitis may well have been caused by the clodronate.

We suspended the immunosuppressive therapy and we planned periodic check-ups. After one year the CT showed a partial reduction of renal ischemia (Figure 1C), even though the chronic renal failure persisted. We did not detect any increase in auto-antibodies or any onset of pathologic events.

## Discussion

In this case, characterized by bilateral kidney necrosis, we made a diagnosis of vasculitis caused by clodronate. The primary diagnostic hypothesis was a renal thrombosis induced by cement embolism after kyphoplasty. In the literature, similar cases following vertebroplasty are reported as being due to cement overflow into paravertebral vessels, eventually reaching the renal artery [13,14]. In our case, this hypothesis was not supported by clinical values, in particular because the coagulation tests were normal. Moreover, unlike in vertebroplasty, cement overflow is rare during kyphoplasty surgery. Furthermore, the bilateral condition is further evidence against the cement overflow hypothesis.

Further clinical exams showed the presence of antibodies, such as anti-ANCA, anti-ds DNA, anti-ANA and anti-MPO. Until this time, the patient had never undergone immunological tests. For this reason, we put forward the hypothesis of immunologic vasculitis caused by a silent form of systemic lupus erythematosus, activated by clodronate. This hypothesis is supported by an experimental study that verified the pro-apoptotic effect of clodronate, as well as its potential autoimmunity induction in SLE mononuclear circulating cells [15]. In accordance with the autoimmunity disease hypothesis, we began immunosuppression therapy that stopped the progression of the disease. The kidney ischemia area and auto-antibody levels decreased.

Further clinical research did not show other SLE clinical signs. Given that drug-induced vasculitis (DIV) could mimic the symptoms of SLE and the spectrum of DIV can range from cutaneous rashes and petechiae or single organ involvement to fatal multi-organ failure with deaths commonly caused by massive pulmonary hemorrhage, we excluded SLE [16]. At this point we hypothesized vasculitis induced by clodronate.

Until now, no cases of DIV caused by clodronate have ever been reported. The offending drug and its metabolites may accumulate within neutrophils, bind to MPO and modify the latter’s configuration which may spread the autoimmune response to other autoantigens and turn them into immunogenic neutrophil proteins (including elastase, lactoferrin and nuclear antigens) [17]. Some drugs could induce neutrophil apoptosis [18]. Neutrophil apoptosis, moreover, in the absence of priming, is associated with translocation of ANCA antigens to the cell surface. This induces the production of ANCAs, which in turn are able to bind to the membrane-bound antigens, causing a self-perpetuating constitutive activation by cross-linking PR3 or MPO and Fcg receptors [19].

There is no standard approach to the treatment of DIV. The first step is the discontinuation of the medication. For patients with severe and active organ failure, intensive immunosuppressive therapy could improve organ function and prevent progression to severe, irreversible disease.

In accordance with the literature, the diagnosis of DIV for our case is defined by the following: (a) the signs and symptoms of vasculitis were temporally related to using the offending drug, and regressed with its discontinuation; (b) blood levels of ANCA antibodies were positive; and (c) medical conditions that mimic vasculitis were excluded, especially malignancies [20].

## Conclusion

We report an adverse case of renal vasculitis induced by clodronate. Considering the ample use on a global scale of this drug for osteoporosis treatment, it is important to highlight the possibility of this adverse effect. Our experience could help in making a quick diagnosis and the timely start of the correct treatment in the future.

## Consent

Written informed consent was obtained from our patient for publication of this case report and accompanying images. A copy of the written consent is available for review by the Editor-in-Chief of this journal.

## Abbreviations

ADP and/or ATP: Adenosine-diphosphate and/or Adenosine-5′-triphosphate; ANA: Anti-nuclear autoantibodies; ANCA: Anti-neutrophil cytoplasmic antibody; anti-ds DNA: Anti-double-stranded DNA antibody; CT: Computed tomography; DEXA: Dual-energy x-ray absorptiometry; DIV: Drug-induced vasculitis; IU: International unit; MPO: Anti-myeloperoxidase antibodies; PMMA: Polymethylmethacrylate cement; SLE: Systemic lupus erythematosus.

## Competing interests

The authors declare that they have no competing interests.

## Authors’ contributions

AN, AC, AP and BM cared for our patient during her hospital stay. AP and LM reviewed our patient’s diagnostic history, signs, laboratory data and investigations, and made the diagnosis. AN and GM wrote the manuscript and performed a literature search. All authors have read, edited and approved the final manuscript.
